# Usefulness of “heart‐shaped sign” during “spaghetti twisting” technique in transvenous lead extraction

**DOI:** 10.1002/joa3.12890

**Published:** 2023-07-02

**Authors:** Takuya Okada, Junji Morita, Yuhei Kasai, Takayuki Kitai, Tsutomu Fujita

**Affiliations:** ^1^ Department of Clinical Engineering, Sapporo Heart Center Sapporo Cardiovascular Clinic Sapporo Japan; ^2^ Department of Cardiology, Sapporo Heart Center Sapporo Cardiovascular Clinic Sapporo Japan

**Keywords:** a tandem femoral‐superior approach, heart‐shaped sign, Needle's Eye Snare, spaghetti twisting technique, transvenous lead extraction

Transvenous lead extraction (TLE) is an indispensable procedure in the management of cardiac implantable electronic devices.^1^ TLE is typically performed via the superior approach. If lead extraction cannot be completed with the superior approach, the femoral vein approach may be necessary to complete the lead extraction.^2^ The Needle's Eye Snare (NES) (Cook Medical Inc.), which consists of a threader and a “cobra head” component, is a useful tool for grasping a lead with inaccessible ends (Figure 1A,B).^3^ The “spaghetti twisting” technique, which enables operators to easily twist and catch a lead, involves using the NES to catch the lead with the cobra head and then passing the threader through the cobra head.^4^ However, one study showed that in some cases, in which deformation is present, the NES cannot grasp the lead even when the NES comes into contact with the targeted lead.^4^ Additionally, an excessive number of attempts to extend the threader of the NES may result in injury to the heart.^5^ We herein describe the appearance of the NES “heart‐shaped sign” and present a useful mechanism for cases in which grasping the lead is difficult. This mechanism can help prevent excessive attempts to deploy the NES, ultimately reducing the risk of complications.

**FIGURE 1:**
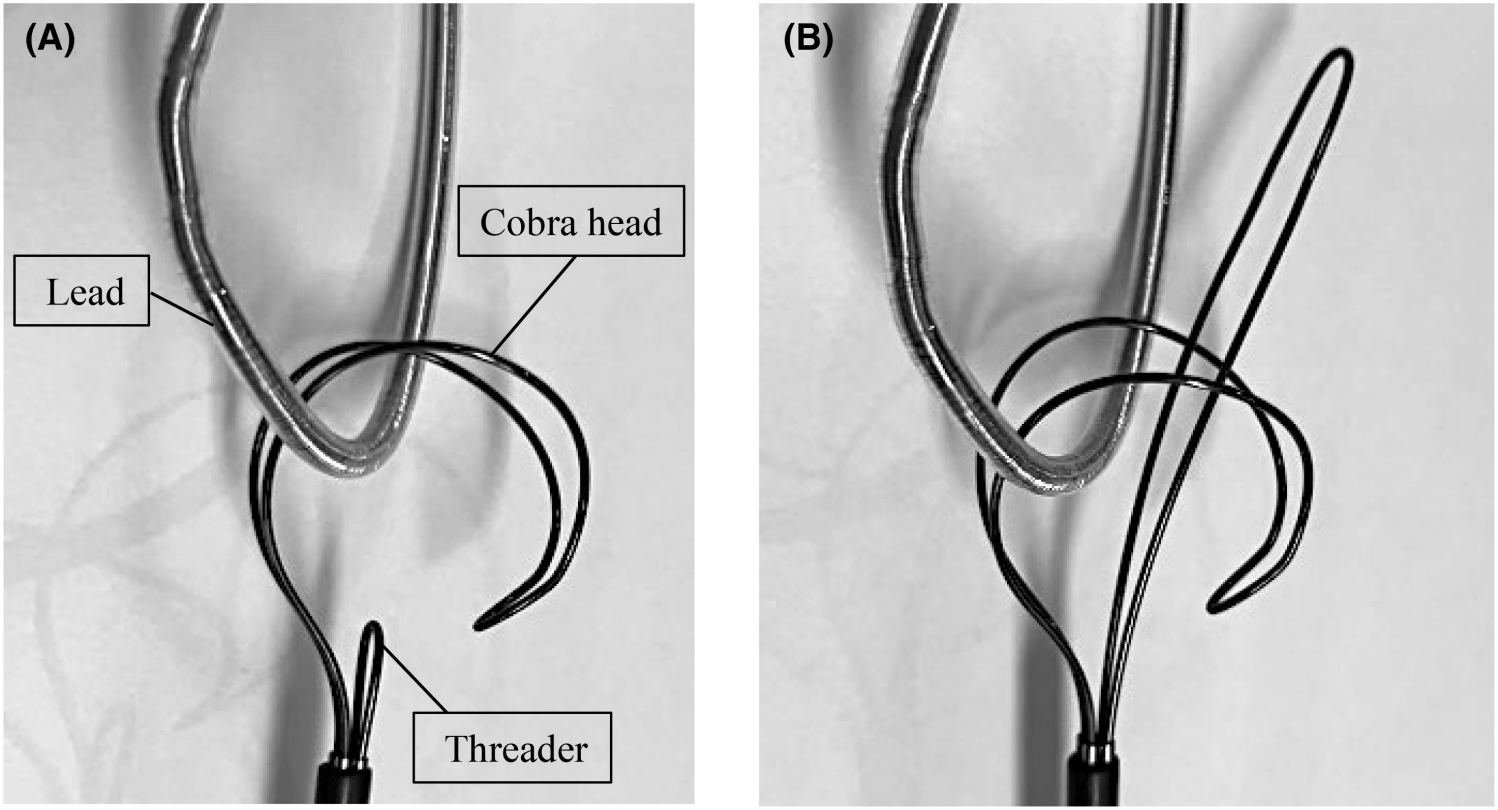
Basic structure of the Needle's Eye Snare (NES). (A) The NES consists of a threader and a cobra head component. (B) Advancing the threader creates a confined space, allowing the lead to be grasped.

A 72‐year‐old man had undergone implantation of a dual‐chamber pacemaker (pulse generator: Azure XT DR MRI SureScan, ventricular lead: 45‐cm CapSure Sense MRI Model 4574, atrial lead: 58‐cm CapSure Fix NOVUS Model 5076; Medtronic) for complete atrioventricular block 13 years previously. He noticed that the lower part of his pacemaker pocket in the left chest had been exposed for 1 month, and he was referred to our hospital to undergo TLE for treatment of pocket infection. After a discussion with the cardiac team, we decided to remove the leads because of device infection. We planned to perform the TLE procedure in a hybrid operating room using general anesthesia with the presence of on‐site cardiac surgery backup. Additionally, we planned to insert the NES from an 18‐Fr long sheath and use the NES to grasp the ventricular lead without a free end. After opening the left pacemaker pocket, we removed the generator and debrided the infected area in the pacemaker pocket. A locking stylet (Liberator; Cook Medical Inc.) was then inserted into the ventricular lead, and an attempt was made to grasp the lead in the right atrium using the NES. However, instead of hooking the lead with the cobra head, the lead got caught on the outside of the cobra (the indentation on the front side) (Figure 2A). In this situation, performing the spaghetti twist technique resulted in a heart‐shaped deformation (Figure 2B,C; Video S1). We, therefore, termed this phenomenon the “heart‐shaped sign.” After hooking the lead with the cobra head, we confirmed that performing a slight twist did not cause the “heart‐shaped sign” to occur, indicating that the lead was properly grasped. After securely grasping the ventricular lead with the NES, the ventricular lead was extracted using a 12‐Fr GlideLight laser sheath (Philips) and an 8.5‐Fr mechanical sheath (Cook Medical, Inc.). During the procedure, the tip of the right atrial lead became detached; therefore, the atrial lead was removed using the 12‐Fr GlideLight laser sheath while being grasped with the Osypka LASSO Snare (Osypka Medtec Inc.) without any complications. One week after the TLE, the patient underwent implantation of a leadless pacemaker (Micra; Medtronic). He was discharged after receiving antibiotic treatment (cefazolin) for 1 month.

**FIGURE 2:**
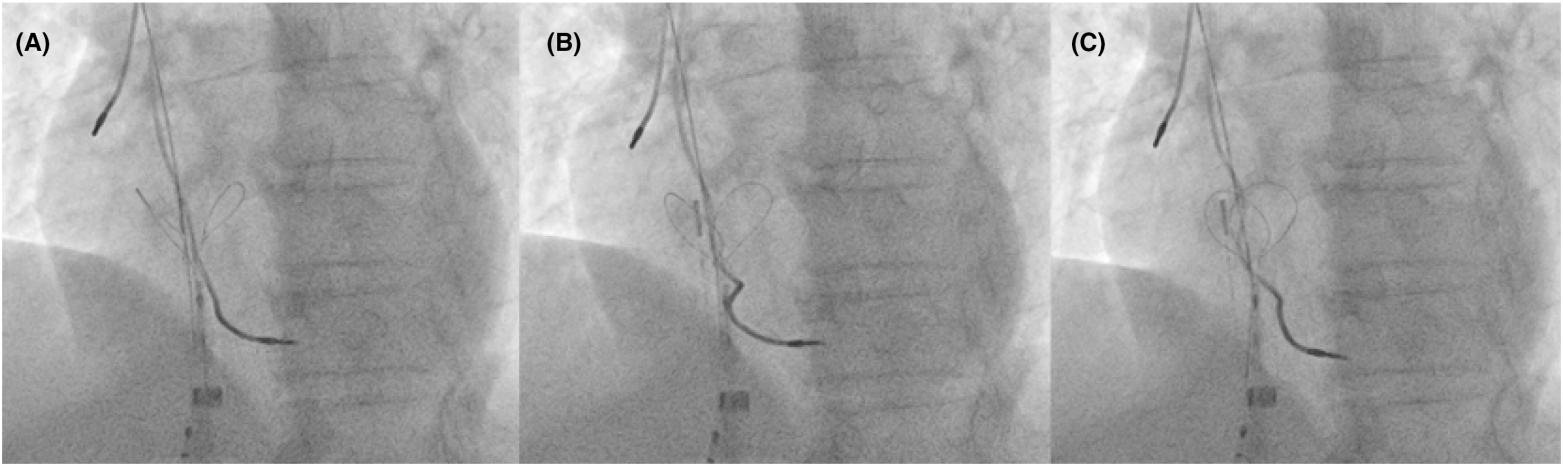
Fluoroscopic images of the transvenous lead extraction procedure. (A) The ventricular lead became trapped on the outer surface of the cobra head component. (B) The Needle's Eye Snare (NES) was rotated. (C) Further rotation of the NES resulted in the cobra head becoming deformed into a heart shape.

A tandem femoral‐superior approach is a useful and safe method for TLE.^2^ The NES is an effective tool for grasping a lead without a free end.^3^ The spaghetti twisting technique using the NES is also a useful method, but failure to grasp the lead may occur despite the fact that it appears to be captured.^4^ We were able to reproduce the heart‐shaped sign in bench tests. In these cases, the lead became caught on an indentation on the front side of the cobra head (Figure 3A), which caused the tool to deform into the heart‐shaped sign when the spaghetti twisting technique was performed (Figure 3B). On the other hand, if the lead caught inside of the cobra head, the spaghetti twisting technique did not result in the formation of the heart‐shaped sign (Figure 3C). The mechanism by which the heart‐shaped sign occurs is that the front of the cobra head moves closer to the back (Figure 4).

**FIGURE 3:**
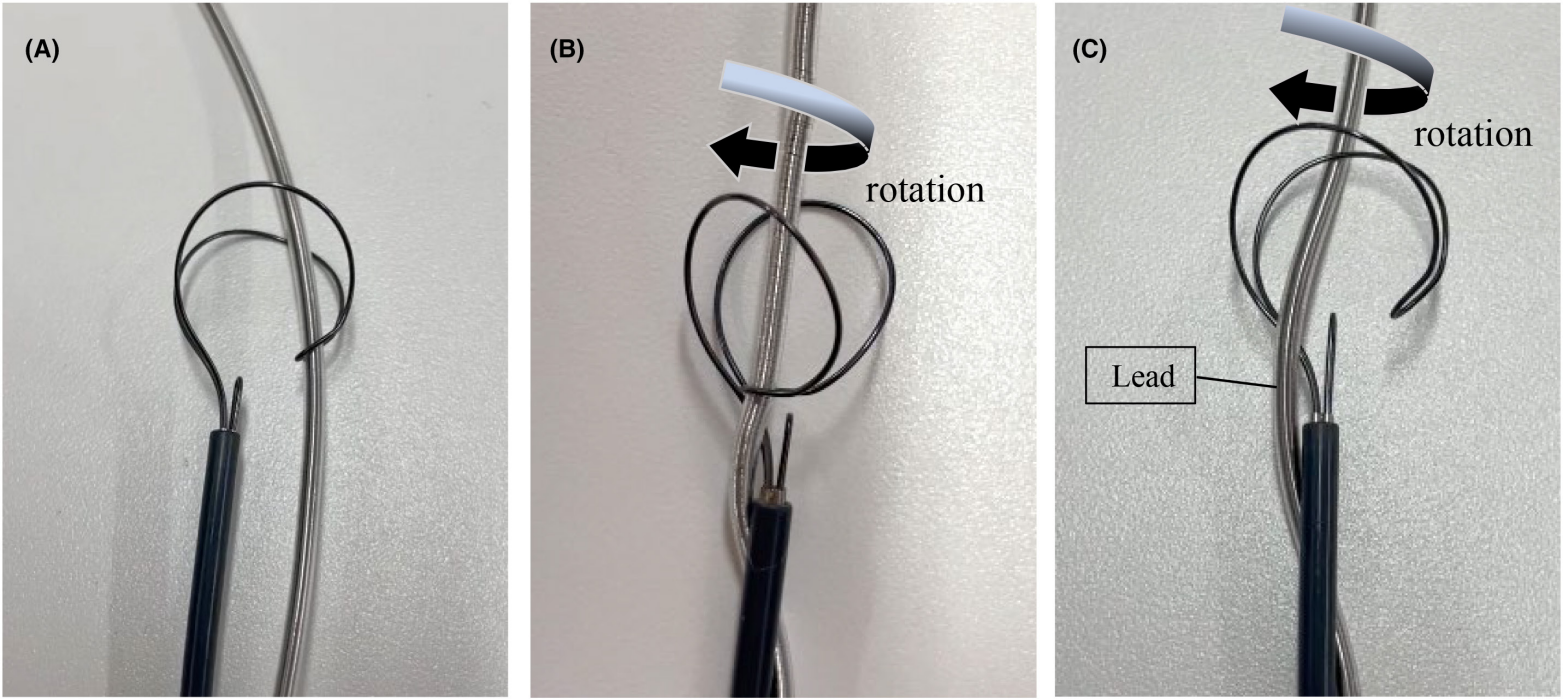
Bench tests of the Needle's Eye Snare. (A) A lead became trapped on an indentation located on the front surface of the cobra head. (B) The front of the cobra head moving closer to the back resulted in the formation of the “heart‐shaped sign.” (C) When the lead was hooked inside the cobra head, the spaghetti twisting technique did not lead to the formation of the “heart‐shaped sign.”

**FIGURE 4:**
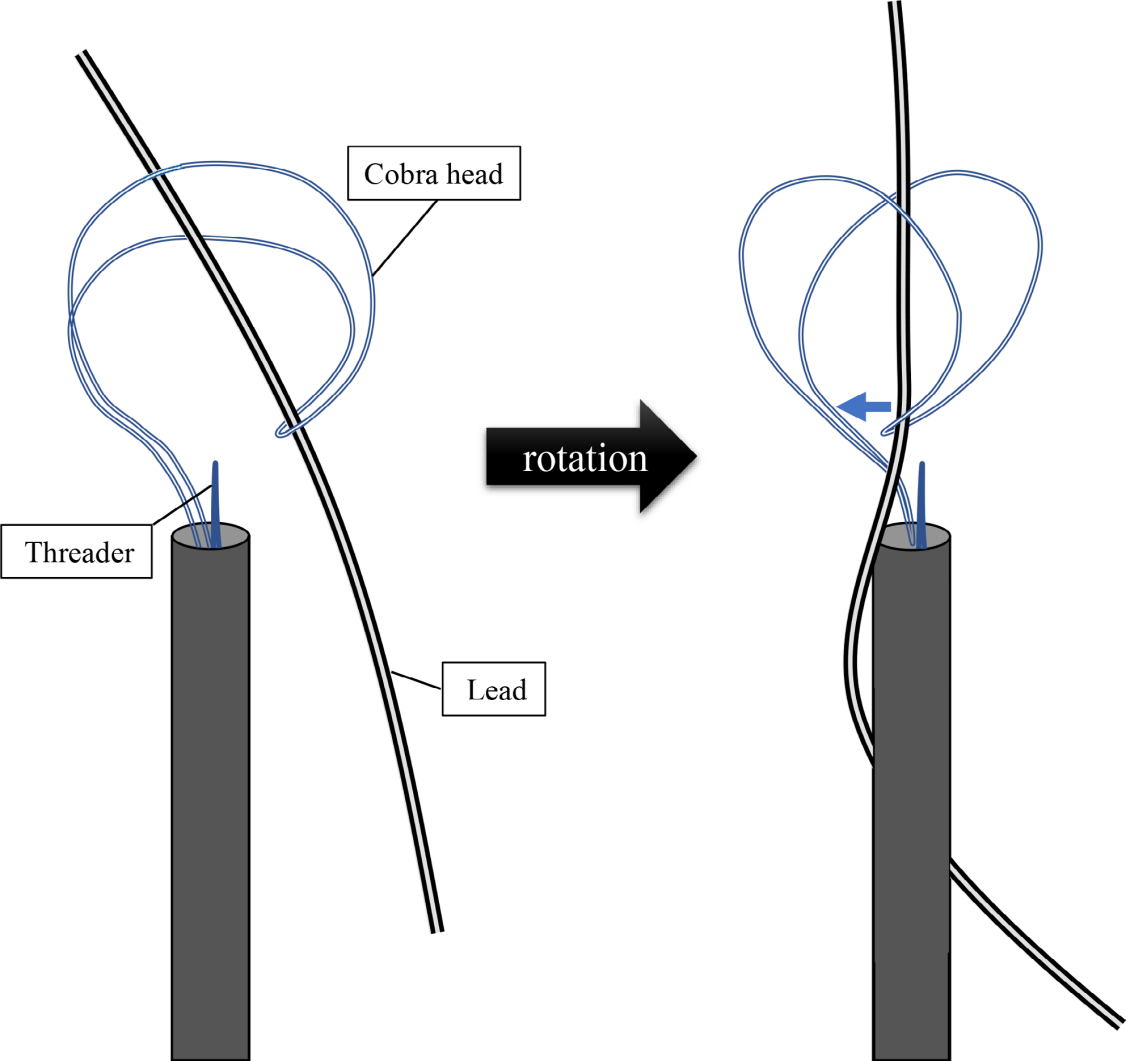
Illustration of the mechanism of the “heart‐shaped sign.”

When the heart‐shaped sign was observed, we confirmed that the lead could not be grasped even with the advancement of the threader, which created no space for closure (Video S2). However, when the cobra head of the NES could hold the lead, the heart‐shaped sign was not reproducible by the spaghetti twisting technique (Video S3). Normally, after the spaghetti twisting technique has been implemented, the threader is advanced to grasp the lead. However, when the heart‐shaped sign is observed, we can decide not to advance the threader again at that point, knowing that the lead cannot be grasped. One case report demonstrated that an excessive number of attempts at threader extension may cause heart injury. By recognizing the heart‐shaped sign, we can become aware of the fact that the lead has not been grasped before the threader is advanced, which can prevent complications beforehand. Furthermore, the heart‐shaped sign provides a clear explanation for the failure to grasp the lead, leading to a reduction in the fluoroscopic and procedure times.

In conclusion, the occurrence of the heart‐shaped sign when performing the spaghetti twisting technique using the NES provides a clear and prompt explanation for the failure to grasp the lead, contributing to a reduction in the risk of complications related to the NES and a decrease in the fluoroscopic and procedure times.

## CONFLICT OF INTEREST STATEMENT

The authors declare no conflict of interests for this article.

## ETHICS STATEMENT

This study was conducted according to the principles of the Declaration of Helsinki. The study was approved by the Institutional Review Board.

## PATIENT CONSENT STATEMENT

The patient provided written informed consent.

## CLINICAL TRIAL REGISTRATION

N/A.

## Supporting information


Video S1.

Video S2.

Video S3.
